# Complete Genome Sequence of *Bradyrhizobium* sp. Strain BDV5419, Representative of Australian Genospecies L

**DOI:** 10.1128/MRA.00042-21

**Published:** 2021-02-25

**Authors:** Christine Oger-Desfeux, Jérôme Briolay, Philippe M. Oger, Bénédicte Lafay

**Affiliations:** a Université de Lyon, Université Claude Bernard-Lyon 1, PRABI-ASMB, FR3728 Bio-Environnement et Santé, Villeurbanne, France; b Université de Lyon, Université Claude Bernard-Lyon 1, CNRS, DTAMB, FR3728 Bio-Environnement et Santé, Villeurbanne, France; c Université de Lyon, INSA de Lyon, CNRS, UMR5240, Villeurbanne, France; d Université de Lyon, École Centrale de Lyon, CNRS, UMR5005, Laboratoire Ampère, Écully, France; e Université de Lyon, Université Claude Bernard-Lyon 1, CNRS, UMR5558, Laboratoire de Biométrie et Biologie Évolutive, Villeurbanne, France; Queens College

## Abstract

We report the complete genome sequence of *Bradyrhizobium* sp. strain BDV5419, representative of *Bradyrhizobium* genospecies L, which symbiotically associates with the Australian native legume *Hardenbergia violaceae* and is expected to represent a novel *Bradyrhizobium* species. The complete genome sequence provides a genetic reference for this Australian genospecies.

## ANNOUNCEMENT

*Bradyrhizobium* strain BDV5419 was isolated in 1995 from a root nodule of *Hardenbergia violaceae* (Fabaceae, Faboideae, Phaseoleae) collected on Black Mountain in Canberra, Australian Capital Territory, Australia (−35°16′1.20″S, 149°05′60.00″E), in the course of a survey of rhizobia associated with native shrubby legumes in southeastern Australia (1). It is the representative strain of *Bradyrhizobium* genospecies L, which may constitute a novel *Bradyrhizobium* species related to Bradyrhizobium elkanii, based on small-subunit (SSU) rRNA gene sequence analysis and phylogeny (1). We sequenced its genome to further the characterization of this genospecies and to gain insight regarding its phylogenetic position and taxonomy within the *Bradyrhizobium* genus.

Strain BDV5419 was grown from a lyophilized stock in 30 ml of yeast extract-mannitol broth (2) at 25°C and 200 rpm for 5 days. Genomic DNA was prepared, as described, by successive phenol-chloroform extractions (3). DNA quantification and quality control were performed using a NanoDrop spectrophotometer, a Qubit 4 fluorometer, and agarose gel electrophoresis. The same DNA was used for Nanopore and Illumina sequencing. Illumina libraries were obtained using the Nextera XT kit following the manufacturer’s instructions, starting with 1 ng of genomic DNA, and were analyzed by paired-end (2 × 300-bp) sequencing on a MiSeq instrument. Poor-quality regions (Q  < 30) of raw reads were removed using Sickle v1.33 (4). Long reads were obtained with an Oxford Nanopore Technologies MinION FLO-MIN106 flow cell from a library prepared with the SQK-RAD004 kit using 1 μg of DNA and 20-s tagmentation. Base calling was performed using Guppy v3.1.5. Sequence quality was assessed using FastQC v0.11.9 (5) for Illumina reads and MinIONQC.R v1.4.2 (6) for Nanopore reads. The preprocessing of Illumina sequencing data using Trimmomatic v0.39 (7) (parameters: ILLUMINACLIP:TruSeq3-PE-2.fa:2:30:10:2:keepBothReads LEADING:3 TRAILING:3 SLIDINGWINDOW:4:15 MINLEN:50) resulted in 3,989,730 paired-end reads (mean lengths of 223 bp and 164 bp for forward and reverse reads, respectively, with ∼200× coverage). Nanopore long reads (total of 2.2 Gbp, with an *N*_50_ value of 33,388 bp) were filtered and trimmed for length (>1,500 bp) and quality (scores of >8) using NanoFilt v2.5.0 (8), and adapters were removed using Porechop v0.2.4 (9). Long reads were further reduced to 800 Mbp as a target quantity using Filtlong v0.2.0 (10) (parameters: --min_length 2000 --keep_percent 90 --target_bases 800000000). Illumina and Nanopore reads were coassembled using Unicycler v.0.4.8 (11) with default parameters. This resulted in a single circular chromosome of length 7,401,610 bp, rotated to start at *dnaA*. Its average G+C content is 64.6%. The assembly was carefully inspected by visualizing the alignment of long and short reads using minimap2 v2.17 (12) and IGV v2.7.2 (13). The genome sequence was automatically annotated by the NCBI Prokaryote Genome Annotation Pipeline (PGAP) v4.13 (14). The genome consists of 6,879 protein-coding genes, 48 tRNAs, 1 copy each of the 5S, 16S, and 23S rRNA genes, and 94 pseudogenes.

### Data availability.

The genome sequence of *Bradyrhizobium* genospecies L strain BDV5419 is available in NCBI GenBank under accession number CP061378. The raw sequence reads are available under SRA accession numbers SRX9514897 and SRX9514899 under BioProject number PRJNA662585 and BioSample number SAMN16089660.
